# Ultra-low-dose coronary CT angiography via super-resolution deep learning reconstruction: impact on image quality, coronary plaque, and stenosis analysis

**DOI:** 10.1007/s00330-025-11399-2

**Published:** 2025-02-01

**Authors:** Li-Miao Zou, Cheng Xu, Min Xu, Ke-Ting Xu, Zi-Cheng Zhao, Ming Wang, Yun Wang, Yi-Ning Wang

**Affiliations:** 1 Department of Radiology, State Key Laboratory of Complex Severe and Rare Diseases, Peking Union Medical College Hospital, Chinese Academy of Medical Sciences and Peking Union Medical College, Beijing, China; 2 Canon Medical System, Beijing, China

**Keywords:** Coronary artery disease, Computed tomography angiography, Radiation dose, Image reconstruction

## Abstract

**Objectives:**

To exploit the capability of super-resolution deep learning reconstruction (SR-DLR) to save radiation exposure from coronary CT angiography (CCTA) and assess its impact on image quality, coronary plaque quantification and characterization, and stenosis severity analysis.

**Materials and methods:**

This prospective study included 50 patients who underwent low-dose (LD) and subsequent ultra-low-dose (ULD) CCTA scans. LD CCTA images were reconstructed with hybrid iterative reconstruction (HIR) and ULD CCTA images were reconstructed with HIR and SR-DLR. The objective parameters and subjective scores were compared. Coronary plaques were classified into three components: necrotic, fibrous or calcified content, with absolute volumes (mm^3^) recorded, and further characterized by percentage of calcified content. The four main coronary arteries were evaluated for the presence of stenosis. Moreover, 48 coronary segments in 9 patients were evaluated for the presence of significant stenosis, with invasive coronary angiography as a reference.

**Results:**

Effective dose decreased by 60% from LD to ULD CCTA scans (2.01 ± 0.84 mSv vs. 0.80 ± 0.34 mSv, *p* < 0.001). ULD SR-DLR was non-inferior or even superior to LD HIR in terms of image quality and showed excellent agreements with LD HIR on the plaque volumes, characterization, and stenosis analysis (ICCs > 0.8). Moreover, there was no evidence of a difference in detecting significant coronary stenosis between the LD HIR and ULD SR-DLR (AUC: 0.90 vs. 0.89; *p* = 1.0).

**Conclusions:**

SR-DLR led to significant radiation dose savings from CCTA while ensuring high image quality and excellent performance in coronary plaque and stenosis analysis.

**Key Points:**

***Question*** *How can radiation dose for coronary CT angiography be reduced without compromising image quality or affecting clinical decisions?*

***Finding*** *Super-resolution deep learning reconstruction (SR-DLR) algorithm allows for 60% dose reduction while ensuring high image quality and excellent performance in coronary plaque and stenosis analysis*.

***Clinical relevance*** *Dose optimization via SR-DLR has no detrimental effect on image quality, coronary plaque quantification and characterization, and stenosis severity analysis, which paves the way for its implementation in clinical practice*.

## Introduction

Coronary CT angiography (CCTA) is a robust imaging modality for noninvasive assessment of obstructive coronary artery disease (CAD) and plaque quantification and characterization, due to its high diagnostic accuracy and wide availability [1, 2]. Moreover, CCTA-derived stenosis and plaque analysis can guide therapeutic decision-making and correlate with patient outcomes [3–5]. Given the broadened use of CCTA in clinical practice, the potential radiation risk remains the main concern [6], and accordingly, various dose-saving strategies have been introduced [7, 8]. However, dose reduction is achieved at the expense of increased image noise and degraded image quality [9], which are critical for further post-processing and interpreting CCTA images.

In this regard, increasing attention has been paid to lowering the radiation burden while maintaining adequate image quality through the use of advanced reconstruction techniques. Iterative reconstruction (IR) algorithms are superior to the traditional filtered back projection (FBP) with lower image noise and better image quality [10, 11]. In recent years, deep learning reconstruction (DLR) algorithms that incorporate deep convolutional neural networks (DCNNs) into image reconstruction have been increasingly utilized. Conventional DLR (C-DLR) algorithms, trained by using a pair of high- and low-noise images, are more efficient in image noise reduction than IR algorithms [12, 13]. Prior studies have validated IR and C-DLR algorithms in granting adequate image quality at low radiation exposure [14–16]. More recently, by utilizing data from ultra-high-resolution (UHR) CT as teaching target, a novel super-resolution DLR (SR-DLR) algorithm has been introduced [17]. Previous studies have reported the noise-reducing effect, improvement of spatial resolution, and better edge sharpness in SR-DLR compared with conventional reconstruction techniques [18–22]. However, whether radiation dose can be effectively reduced by SR-DLR remains unknown.

Therefore, the aim of this study was to exploit the dose reduction capability of SR-DLR and assess its impact on image quality, plaque quantification and characterization, and stenosis severity analysis.

## Materials and methods

### Study participants

The protocol for this prospective study was reviewed and approved by the local ethics committee and written informed consent was obtained from all patients.

From December 2023 to May 2024, patients who were referred for the assessment of known or suspected CAD with CCTA were consecutively enrolled. The exclusion criteria were as follows: age < 18 years, allergic reactions to iodinated contrast medium and renal insufficiency. Pre-test probability of CAD was calculated based on age, sex, and symptoms, according to the 2019 ESC guidelines for the diagnosis and management of chronic coronary syndromes [23].

### CT acquisition and image reconstruction

In all patients, a noncontrast cardiac-gated CT scan for coronary artery calcium scoring followed by two sequential one-beat CCTA exams at low dose (LD) and ultra-low-dose (ULD) were performed on a 320-row detector CT scanner (Aquilion ONE GENESIS, Canon Medical Systems). All patients received sublingual nitroglycerin (1.0 mg) 1–2 min before examinations and for patients with heart rate ≥ 75 beats/min, additional β blockers were administered. The contrast agent (Iopamiro, 370 mgI/mL; Bracco Sine Pharma) was injected at a rate of body weight (kg) × 0.053 mL/s in 10 s (fixed), followed by 30 mL of saline at the same injection rate. Bolus tracking with a region of interest (ROI) in the descending aorta was used for scan initiation, with a trigger threshold of 280 HU. For the LD scan, the tube current was adjusted automatically based on a noise index (SD) of 38 and the ULD scan was performed with 40% of the tube current used for the LD scan (i.e., according to a prior study [24], SR-DLR achieved a noise reduction of up to 55% compared to hybrid IR (HIR) and may theoretically allow for 60% tube current reduction). Other acquisition parameters remained consistent for the two sequential scans: a tube voltage of 100 kVp; a gantry rotation time of 0.275 s; collimation of 320 × 0.5 mm.

The CT dose index volume (CTDIvol, mGy) and dose-length product (DLP, mGy·cm) were recorded. The estimated effective dose (ED, mSv) was calculated as DLP multiplied by the conversion factor (0.026 mSv·mGy^−1^·cm^−1^) [25]. CTDIvol, DLP, and ED were compared between LD and ULD scans.

LD CCTA images were reconstructed with HIR (Adaptive Iterative Dose Reduction 3D [AIDR 3D], Canon Medical Systems) and ULD CCTA images were reconstructed with HIR and SR-DLR (Precise IQ Engine [PIQE], Canon Medical Systems). Reconstruction kernels were as follows: FC43 kernel for HIR, Cardiac kernel for SR-DLR. All the reconstruction datasets had a slice thickness of 0.5 mm with 0.25-mm intervals.

### Objective image quality evaluation

A board-certified radiologist (with 3 years of clinical practice experience in cardiac radiology) performed objective image analysis. All CCTA datasets were simultaneously displayed on the screen, with ROIs placed at exactly the same locations.

#### Image noise, signal-to-noise ratio (SNR) and contrast-to-noise ratio (CNR)

The mean CT attenuation (in HU) was measured in the aorta root, the proximal segment of main coronary arteries (left main trunk, left anterior descending artery, left circumflex and right coronary artery), as well as adjacent adipose tissue. The ROIs were carefully chosen to avoid the vessel wall, calcification and stent. Image noise was defined as the standard deviation (SD) of CT attenuation. The SNR was determined by dividing the CT attenuation by the image noise. The CNR was determined by dividing the difference in CT attenuation between the vessel lumen and surrounding adipose tissue by image noise.

#### Edge sharpness and full width at half maximum (FWHM)

We generated CT attenuation profiles of coronary arteries, calcifications, and stents by setting a linear ROI on a cross-sectional image using ImageJ software (https://imagej.net/ij/), using the definitions introduced before [19]. Then the edge sharpness and FWHM calculations were performed with in-house code developed in Matlab (version R2019a). Edge sharpness was examined on both sides of the profiles and the mean values were recorded for the following analysis.

### Subjective image quality evaluation

Subjective image quality was independently evaluated by two board-certified radiologists (with 7 and 3 years of clinical practice experience in cardiac radiology, respectively) in a blinded fashion using a 5-point Likert scale. Images were rated according to the severity of image noise, vessel attenuation, quality of contour delineation, and general image impression (1 = poor; 2 = fair; 3 = moderate; 4 = good; 5 = excellent).

### Coronary plaque quantification and characterization

**Fig. 1:**
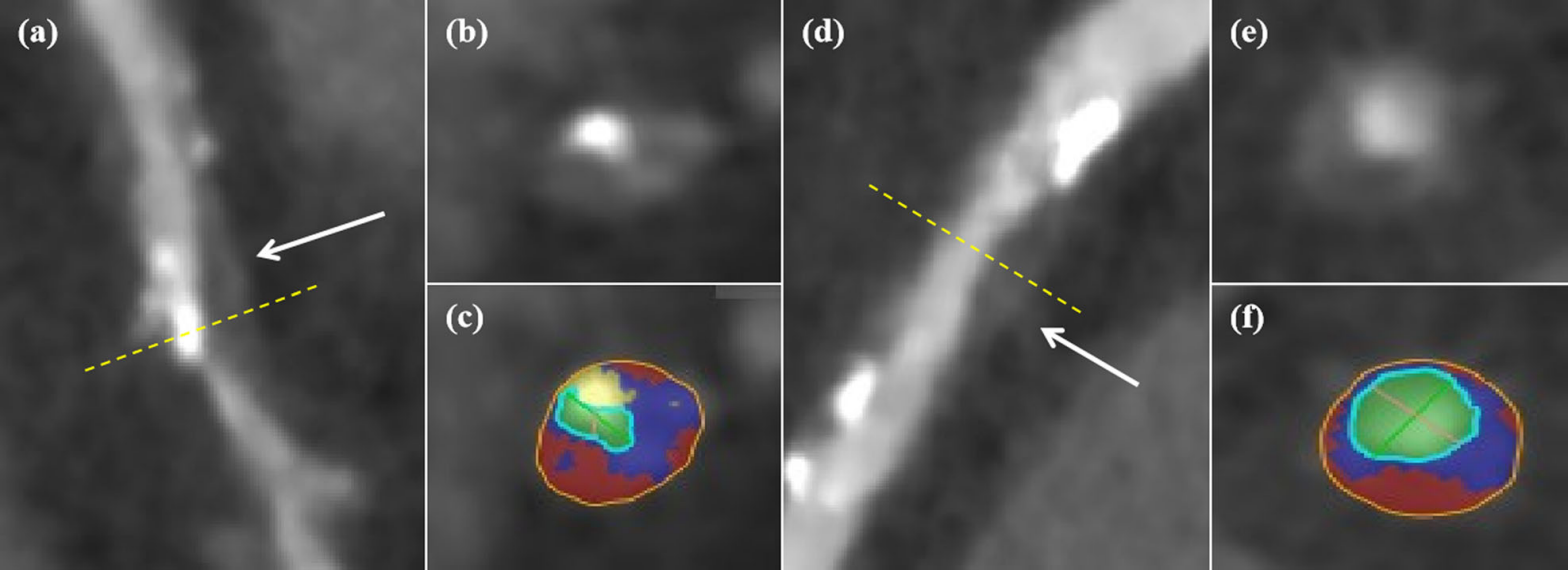
Case examples of CT attenuation values-based coronary plaque classification. Curved multiplanar reformation and axial images of a mixed plaque (**a**–**c**) and a non-calcified plaque (**d**–**f**) are shown. Lumen area (green), necrotic content (red), fibrous content (blue) and calcified content (yellow)

All the datasets were transferred to a dedicated Vitrea workstation (version 7.6) for plaque analysis. Between the outer vessel wall boundary and the luminal boundary, coronary plaques were classified into three components by user-defined, fixed HU threshold values: necrotic content (< 30 HU), fibrous content (30–350 HU) or calcified content (> 350 HU) [26] (Fig. 1). Absolute volumes (in mm^3^) of the three components and total plaque were computed. The percentage of calcified content was defined as the volume of calcified content divided by total plaque volume × 100%. Plaques were further characterized by the percentage of calcified content: non-calcified plaque (< 20%), mixed plaque (20–80%), and calcified plaque (> 80%).

### Assessment of coronary artery stenosis

CCTA images were visually evaluated by two board-certified radiologists who were blinded to the clinical information of patients in a joint reading and any disagreements were resolved by consensus.

For each patient, the four main coronary arteries (left main trunk, left anterior descending artery, left circumflex and right coronary artery) were evaluated for the presence of stenosis and categorized as: 1–24% (minimal stenosis), 25–49% (mild stenosis), 50–69% (moderate stenosis), 70–99% (severe stenosis), and 100% (total occlusion). Based on the most severe stenosis detected on CCTA, per-patient CAD-Reporting and Data System (CAD-RADS) categories were determined. For patients who underwent invasive coronary angiography (ICA), the coronary arteries were divided into 18 segments based on the Society of Cardiovascular Computed Tomography classification and coronary arteries with a diameter of ≥ 1.5 mm were evaluated for the presence of significant coronary artery stenosis (> 50%).

### Invasive coronary angiography

Clinically indicated ICA was performed using an Allura Xper UNIQ FD10 system (Philips Medical Systems). Images were acquired through multiple projections and at least two orthogonal projections were interpreted for > 50% stenosis by two experienced cardiologists in consensus, using the same 18-segment model.

### Statistical analysis

Statistical analyses were conducted using R software (version 3.6.1; http://www.R-project.org). The Shapiro-Wilk test was employed to assess the normal distribution of the data. Quantitative parameters were presented as mean values ± SD or median (interquartile range), while qualitative parameters were expressed as frequencies and composition ratios (%). For data with a normal distribution, one-way repeated analysis of variance (ANOVA) was used, and paired samples *t*-tests with Bonferroni correction were used for multiple comparisons. For data that were not normally distributed, the Friedman test was used, followed by the Wilcoxon signed-rank test with Bonferroni correction for subsequent multiple comparisons. Interobserver agreement for subjective evaluations was assessed using kappa coefficients, with the following interpretation: ≤ 0.40 indicates poor agreement; 0.41–0.60 indicates moderate agreement; 0.61–0.80 indicates good agreement; > 0.80 indicates excellent agreement. Intraclass correlation (ICC) analysis was conducted to evaluate the reliability of plaque assessment, stenosis severity analysis, and per-patient CAD-RADS category between LD and ULD scans. Using ICA as the reference standard, the diagnostic performance metrics—including sensitivity, specificity, negative predictive value (NPV), positive predictive value (PPV), diagnostic accuracy, and area under the receiver operating characteristic curve (AUC)—were compared. The R package “DTComPair” (https://cran.r-project.org/web/packages/DTComPair/index.html) was used to compute *p*-values for the binary diagnostic tests, while AUCs were compared using the DeLong method. Bonferroni corrections were applied for multiple comparisons, and a *p*-value < 0.05 was considered statistically significant.

## Results

### Patient characteristics and radiation exposure

**Fig. 2:**
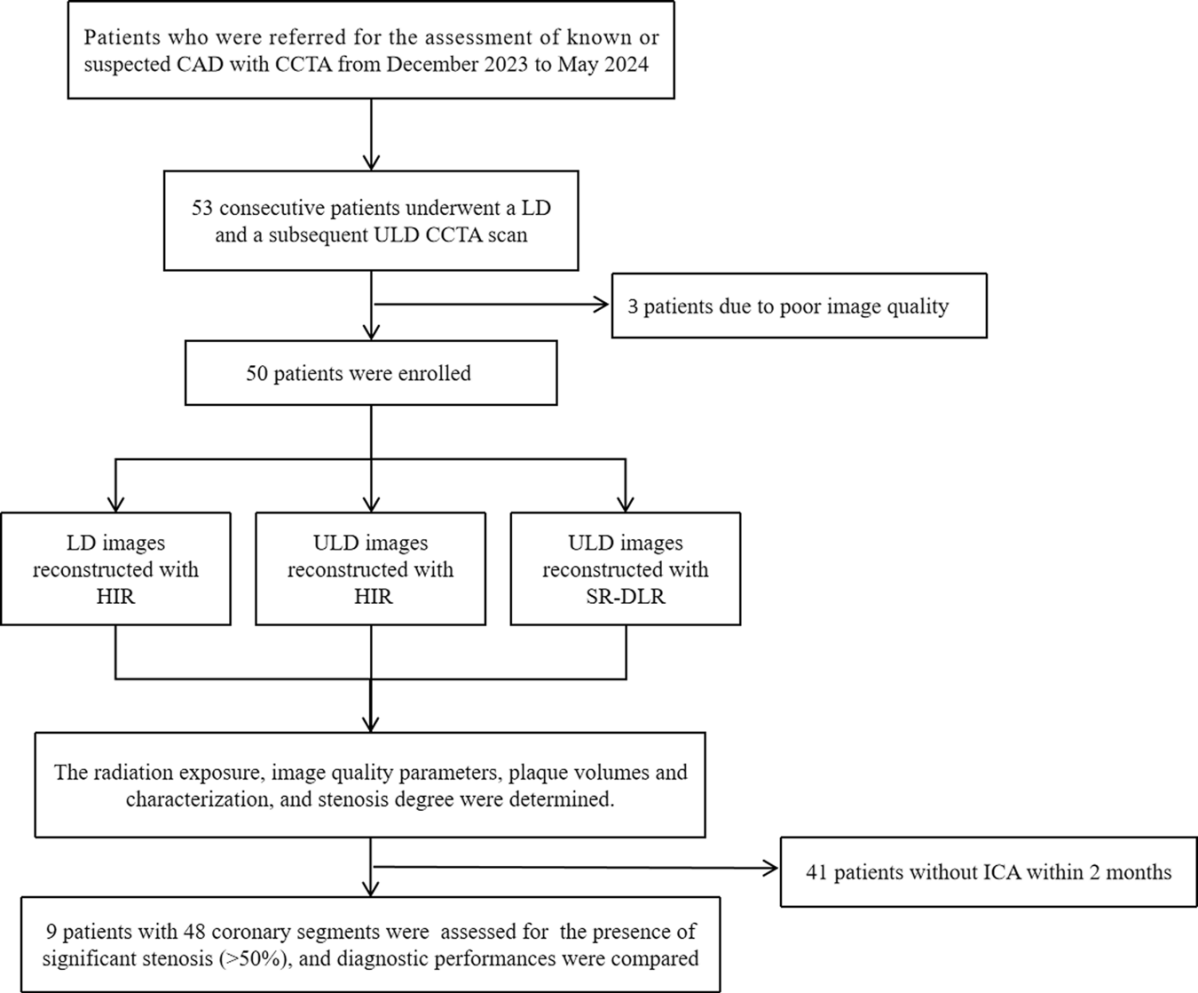
Flowchart of patient inclusion and study design. CAD, coronary artery disease; CCTA, coronary CT angiography; LD, low dose; ULD, ultra-low-dose; HIR, hybrid iterative reconstruction; SR-DLR, super-resolution deep learning reconstruction; ICA, invasive coronary angiography

Fifty-three patients underwent a LD and a subsequent ULD CCTA scan, and three patients were excluded because of poor image quality. Finally, 50 patients (mean age, 62.9 years ± 11.2 [SD]; 35 men, 15 women) were included in this study, of which 9 patients underwent ICA within 2 months as a reference standard. The mean pre-test probability was 22.4% ± 11.8% (ranging from 6 to 52%). The CTDIvol, DLP and ED of ULD scans were significantly lower than those of LD scans (1.97 ± 0.80 mGy vs. 4.92 ± 2.01 mGy; 30.87 ± 12.92 m Gy·cm vs. 77.17 ± 32.30 mGy·cm; 0.80 ± 0.34 mSv vs. 2.01 ± 0.84 mSv, all *p* < 0.001). The characteristics of 50 patients are specified in Table 1. Figure 2 shows the flowchart of patient inclusion and study design.

**Table 1:** Detailed patient baseline characteristics

Characteristic	Value (*n* = 50)
Sex	
Male	35 (70%)
Female	15 (30%)
Age (years)	62.9 ± 11.2
Body mass index (kg/m^2^)	26.0 ± 3.1
Height (m)	1.68 ± 0.06
Weight (kg)	73.3 ± 10.2
Risk factors for CAD	
Diabetes (%)	25 (50%)
Hypertension (%)	32 (64%)
Hypercholesterolemia (%)	18 (36%)
Smoking (%)	28 (56%)
CAD family history (%)	14 (28%)
Symptoms	
Typical angina (%)	16 (32%)
Atypical angina (%)	11 (22%)
Non-anginal chest pain (%)	10 (20%)
Dyspnea (%)	13 (26%)
Agatston score	209.90 [28.83, 531.93]

Data are presented as numbers (percentages), mean ± standard deviation, or median (IQR)

*CAD* coronary artery disease, *IQR* interquartile range

### Objective image quality evaluation

**Fig. 3:**
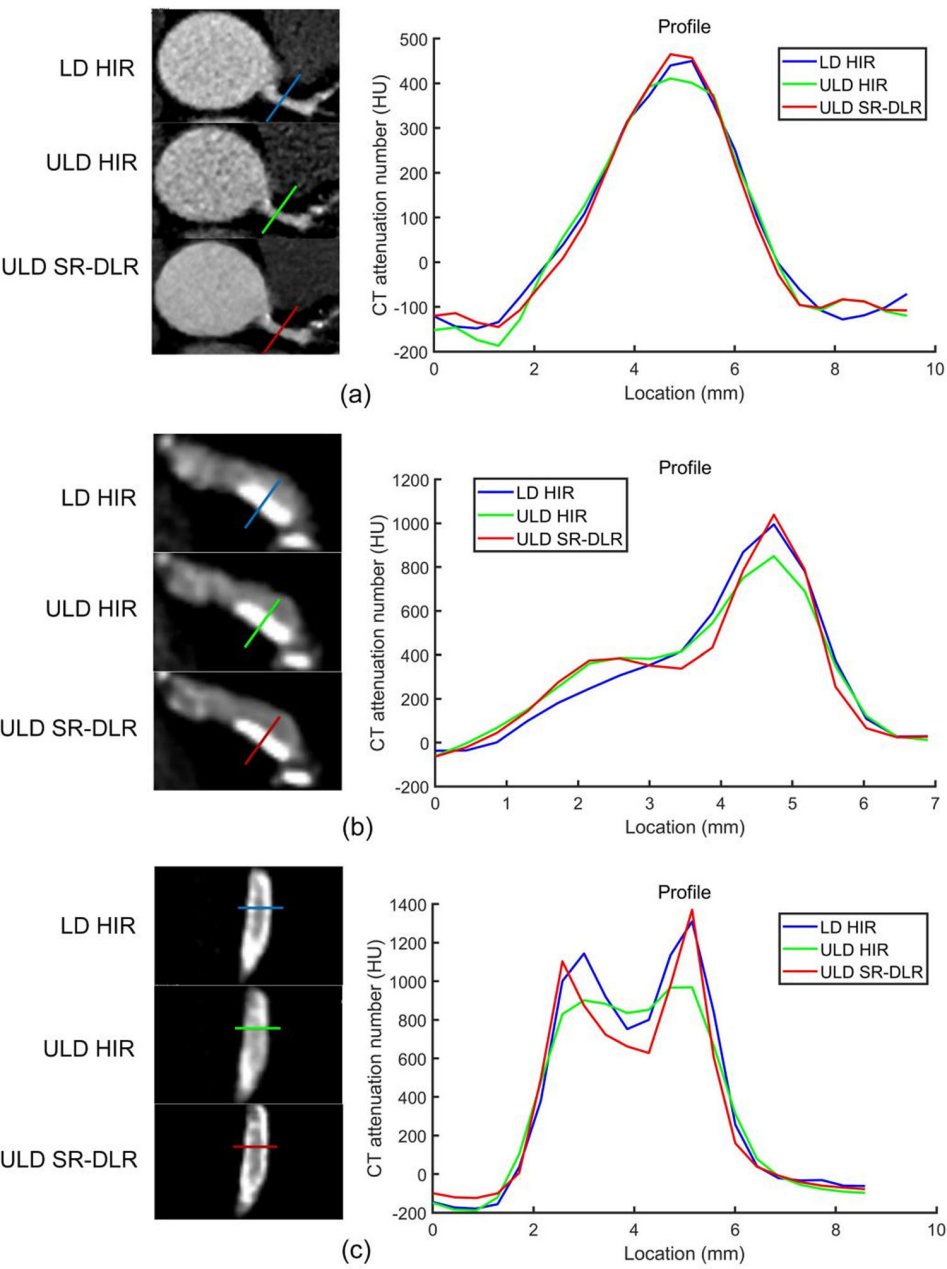
Profile curves of coronary arteries (**a**), calcifications (**b**), and stents (**c**). Edge sharpness is defined as the maximum slope of the profile curve. FWHM represents full width at half maximum. LD, low dose; ULD, ultra-low dose; HIR, hybrid iterative reconstruction; SR-DLR, super-resolution deep learning reconstruction

ULD SR-DLR outperformed ULD HIR regarding the image noise, SNR and CNR, edge sharpness, and FWHM of coronary arteries, calcifications, and stents (all *p* < 0.001). LD HIR was superior to ULD HIR with regard to image noise, SNR, CNR, edge sharpness of coronary arteries, calcifications, and stents (all *p* < 0.05). FWHM of coronary arteries was smaller with LD HIR than with ULD HIR (*p* < 0.001) while FWHM of calcifications did not differ (*p* = 0.42). FWHM-lumen calculated on LD HIR (1.29 mm ± 0.36) was larger than on ULD HIR (1.10 mm ± 0.52; *p* < 0.001) whereas FWHM-stent was thinner on LD HIR than on ULD HIR, but not statistically significant (0.90 mm ± 0.24 vs. 1.24 mm ± 0.96; *p* = 0.93). The image noise, CNR and SNR of ULD SR-DLR were comparable or superior to those of LD HIR. Edge sharpness and FWHM of coronary arteries did not differ (all *p* > 0.05), while edge sharpness of calcifications was significantly higher and FWHM was smaller with ULD SR-DLR than with LD HIR (all *p* < 0.001). Moreover, edge sharpness of stents was 90% higher (440.82 HU/mm ± 277.83 vs. 232.44 HU/mm ± 161.10; *p* < 0.001) with ULD SR-DLR than with LD HIR. ULD SR-DLR had a larger FWHM of the lumen (1.69 mm ± 0.46 vs. 1.29 mm ± 0.36; *p* < 0.001) and smaller strut (0.83 mm ± 0.18 vs. 0.90 mm ± 0.24; *p* < 0.05) than LD HIR. Detailed results are given in Table 2 and Fig. 3.

**Table 2:** Objective image quality assessment

Parameter	LD HIR	ULD HIR	ULD SR-DLR	*p*-values			
				All	LD HIR vs. ULD HIR	LD HIR vs. ULD SR-DLR	ULD HIR vs. ULD SR-DLR
Noise							
Ao	34.10 (4.38)	41.97 (5.03)	19.31 (3.05)	< 0.001	< 0.001	< 0.001	< 0.001
LM	24.68 (8.56)	31.97 (13.35)	20.11 (11.00)	< 0.001	< 0.001	< 0.001	< 0.001
LAD	26.01 (7.52)	34.74 (13.95)	21.94 (8.27)	< 0.001	< 0.001	0.008	< 0.001
LCX	24.77 (11.24)	30.44 (16.37)	24.12 (19.52)	< 0.001	< 0.001	0.18	< 0.001
RCA	24.32 (7.12)	30.26 (11.40)	18.87 (9.44)	< 0.001	< 0.001	< 0.001	< 0.001
SNR							
Ao	12.44 (2.42)	9.40 (2.32)	22.05 (5.95)	< 0.001	< 0.001	< 0.001	< 0.001
LM	19.32 (8.87)	14.98 (10.36)	23.63 (13.60)	< 0.001	< 0.001	0.05	< 0.01
LAD	17.06 (6.36)	12.89 (8.64)	19.60 (15.25)	< 0.001	< 0.001	1.00	< 0.001
LCX	19.65 (8.53)	15.20 (7.36)	22.22 (13.34)	< 0.001	< 0.001	0.627	< 0.001
RCA	18.11 (6.39)	14.45 (7.90)	24.58 (17.88)	< 0.001	< 0.001	< 0.001	< 0.001
CNR							
Ao	17.44 (4.95)	13.40 (3.64)	26.19 (7.38)	< 0.001	< 0.001	< 0.001	< 0.001
LM	18.76 (7.66)	13.43 (3.59)	22.63 (7.31)	< 0.001	< 0.001	0.008	< 0.001
LAD	20.05 (6.68)	14.37 (4.33)	23.57 (7.12)	< 0.001	< 0.001	0.06	< 0.001
LCX	21.16 (8.27)	14.26 (4.36)	24.24 (8.21)	< 0.001	< 0.001	0.008	< 0.001
RCA	18.37 (5.88)	13.12 (3.69)	22.61 (7.11)	< 0.001	< 0.001	< 0.001	< 0.001
Vessel (*n* = 50)							
Edge sharpness (HU/mm)	229.66 (77.86)	200.97 (72.74)	218.79 (102.28)	< 0.001	< 0.001	0.15	< 0.001
FWHM (mm)	3.85 (0.79)	4.05 (0.74)	3.87 (0.81)	0.003	0.01	1.00	< 0.001
Calcification (*n* = 39)							
Edge sharpness (HU/mm)	594.32 (173.21)	543.32 (182.79)	699.31 (245.20)	< 0.001	0.003	< 0.001	< 0.001
FWHM (mm)	2.93 (0.93)	3.02 (0.83)	2.51 (0.90)	< 0.001	0.42	< 0.001	< 0.001
Stent (*n* = 60)							
Edge sharpness (HU/mm)	232.44 (161.10)	177.74 (136.44)	440.82 (277.83)	< 0.001	< 0.001	< 0.001	< 0.001
FWHM (mm)	0.90 (0.24)	1.24 (0.96)	0.83 (0.18)	< 0.001	0.93	0.04	< 0.001
FWHM-lumen	1.29 (0.36)	1.10 (0.52)	1.69 (0.46)	< 0.001	< 0.001	< 0.001	< 0.001

Data are presented as mean ± standard deviation

*LD* low dose, *ULD* ultra-low-dose, *HIR* hybrid iterative reconstruction, *SR-DLR* super-resolution deep learning reconstruction, *Ao* aortic root, *LM* left main artery, *LAD* left anterior descending artery, *LCX* left circumflex, *RCA* right coronary artery, *SNR* signal-to-noise ratio, *CNR* contrast-to-noise ratio, *FWHM* full width at half maximum

### Subjective image quality evaluation

The subjective image quality scores for LD HIR, ULD HIR and ULD SR-DLR were 3.30, 2.52, and 4.42 for reader 1 and 3.32, 2.48, and 4.54 for reader 2, respectively (Supplementary material, Table 1). There was excellent interobserver agreement with respect to the overall image quality (κ = 0.837). According to the scores, the subjective image quality of ULD SR-DLR was superior to that of LD HIR; followed by ULD HIR.

### Coronary plaque quantification and characterization

**Fig. 4:**
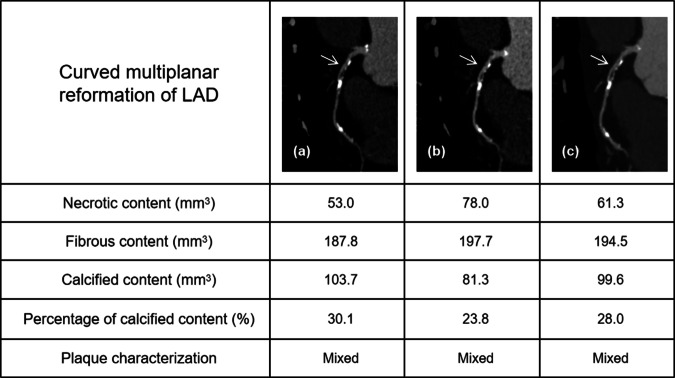
Case example. An 81-year-old patient with a mixed plaque at the proximal segment of LAD. Curved multiplanar reformation of the analyzed plaque of LD HIR (**a**), ULD HIR (**b**), ULD SR-DLR (**c**) is shown. LAD, left anterior descending artery; LD, low dose; HIR, hybrid iterative reconstruction; ULD, ultra-low-dose; SR-DLR, super-resolution deep learning reconstruction

Agreements on absolute volumes were excellent between LD HIR and ULD SR-DLR for all components, with ICCs of 0.909 (95% CI, 0.850–0.946), 0.973 (95% CI, 0.955–0.984), 0.980 (95% CI, 0.946–0.991) and a mean difference of 1.39 mm^3^ (± 5.91mm^3^), −0.55 mm^3^ (± 8.03mm^3^), −4.74 mm^3^ (± 8.07mm^3^) for necrotic content, fibrous content, and calcified content, respectively. Agreements on absolute volumes were also excellent between LD HIR and ULD HIR, with ICC and a mean difference of 0.909 (95% CI, 0.832–0.949), 2.35 mm^3^ (± 5.71mm^3^) for necrotic content; 0.919 (95% CI, 0.866–0.951), 1.95 mm^3^ (± 13.81 mm^3^) for fibrous content; 0.958 (95% CI, 0.931–0.975), −2.58 mm^3^ (± 14.27mm^3^) for calcified content, respectively. Reliability of plaque characterization was excellent for LD HIR and ULD SR-DLR and good for LD HIR and ULD HIR, with ICCs of 0.870 (95% CI, 0.790–0.922) and 0.763 (95% CI, 0.625–0.854). Detailed results are given in Table 3, and Fig. 4 shows the example case.

**Table 3:** Coronary plaque quantification and characterization

	LD HIR vs. ULD HIR	LD HIR vs. ULD SR-DLR
Necrotic content volume (HU < 30)		
ICC (95% CI)	0.909 (0.832, 0.949)	0.909 (0.850, 0.946)
Mean bias ± SD	2.35 mm^3^ ± 5.71	1.39 mm^3^ ± 5.91
Fibrous content volume (HU: 30 ~ 350)		
ICC (95% CI)	0.919 (0.866, 0.951)	0.973 (0.955, 0.984)
Mean bias ± SD	1.95 mm^3^ ± 13.81	−0.55 mm^3^ ± 8.03
Calcified content volume (HU > 350)		
ICC (95% CI)	0.958 (0.931, 0.975)	0.980 (0.946, 0.991)
Mean bias ± SD	−2.58 mm^3^ ± 14.27	−4.74 mm^3^ ± 8.07
Plaque characterization		
ICC (95% CI)	0.763 (0.625, 0.854)	0.870 (0.790, 0.922)

*LD* low dose, *ULD* ultra-low dose, *HIR* hybrid iterative reconstruction, *SR-DLR* super-resolution deep learning reconstruction, *ICC* intraclass correlation coefficient

### Coronary artery stenosis analysis

ICCs for stenosis analysis were 0.973 (95% CI, 0.959–0.982) between LD HIR and ULD SR-DLR, and 0.829 (95% CI, 0.670–0.903) between LD HIR and ULD HIR. Table 4 shows the agreement on stenosis analysis. As for per-patient CAD-RADS category, ICCs were 0.983 (95% CI, 0.968–0.991) between LD HIR and ULD SR-DLR and 0.838 (95% CI, 0.661–0.926) between LD HIR and ULD HIR (Supplementary material, Table 2).

**Table 4:** Agreement on stenosis analysis

A. Agreement on stenosis analysis between LD HIR and ULD HIR					
	LD HIR				
ULD HIR	1–24%	25–49%	50–69%	70–99%	100%
1–24%	4	2	0	0	0
25–49%	5	21	0	0	0
50–69%	0	16	28	0	0
70–99%	0	0	8	18	0
100%	0	0	0	0	2

B. Agreement on stenosis analysis between LD HIR and ULD SR-DLR					
	LD HIR				
ULD SR-DLR	1–24%	25–49%	50–69%	70–99%	100%
1–24%	9	0	0	0	0
25–49%	0	36	0	0	0
50–69%	0	3	34	0	0
70–99%	0	0	2	18	0
100%	0	0	0	0	2

*LD* low dose, *ULD* ultra-low-dose, *HIR* hybrid iterative reconstruction, *SR-DLR* super-resolution deep learning reconstruction

**Fig. 5:**
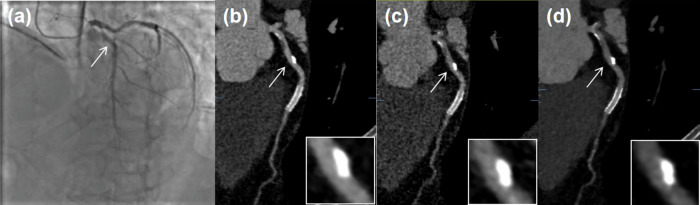
A representative case of a 71-year-old patient with a calcified plaque at the proximal segment of LAD. **a** In ICA, no significant stenosis was found. Curved multiplanar reformations of LD HIR-(**b**), ULD HIR-(**c**), ULD SR-DLR-(**d**) showed a stenosis degree of < 50%, > 50%, < 50%, respectively. LAD, left anterior descending artery; ICA, invasive coronary angiography; LD, low dose; HIR, hybrid iterative reconstruction; ULD, ultra-low dose; SR-DLR, super-resolution deep learning reconstruction

The accuracy of stenosis analysis was validated in nine patients who underwent ICA. In total, 48 coronary segments were assessed for the presence of significant stenosis and ICA showed > 50% stenosis in 25 out of them (52%). Using ICA as the reference standard, the diagnostic performance of LD HIR was as follows: sensitivity = 92%, specificity = 87%, PPV = 89%, NPV = 91%, accuracy = 90%, AUC = 0.90. The corresponding diagnostic performance measurements were 96%, 83%, 86%, 95%, 90%, 0.89 for ULD SR-DLR and 92%, 52%, 68%, 86%, 73%, 0.72 for ULD HIR, respectively. There was no evidence of a difference in all diagnostic parameters between the LD HIR and ULD SR-DLR (all *p* > 0.05). Both LD HIR and ULD SR-DLR outperformed ULD HIR with regard to specificity, PPV, accuracy, and AUC (all *p* < 0.05) while sensitivity and NPV remained unchanged (all *p* > 0.05). Table 5 shows the detailed diagnostic performances, and Fig. 5 shows the representative case.

**Table 5:** Diagnostic accuracy of CCTA for the detection of significant coronary stenosis

	LD HIR	ULD HIR	ULD SR-DLR	*p*-values		
				LD HIR vs. ULD HIR	LD HIR vs. ULD SR-DLR	ULD HIR vs. ULD SR-DLR
TP	23	23	24	/	/	/
TN	20	12	19	/	/	/
FP	3	11	4	/	/	/
FN	2	2	1	/	/	/
Sensitivity (%)	92 (80, 100)	92 (80, 100)	96 (87, 100)	1.00	0.95	1.00
Specificity (%)	87 (72, 100)	52 (32, 73)	82 (65, 96)	0.01	0.95	0.02
PPV (%)	88 (75, 100)	68 (51, 83)	86 (72, 97)	0.01	1.00	0.02
NPV (%)	91 (77, 100)	86 (64, 100)	95 (83, 100)	1.00	1.00	0.99
Accuracy (%)	90 (81, 98)	73(60, 85)	90 (81, 98)	0.02	1.00	0.02
AUC	0.90 (0.81, 0.98)	0.72 (0.60, 0.84)	0.89 (0.81, 0.98)	0.01	1.00	0.01

The values for sensitivity, specificity, PPV, NPV and accuracy are presented with 95% CIs

Bonferroni correction were applied for multiple comparisons, *p* < 0.05 was considered statistically significant

*LD* low dose, ULD ultra-low-dose, *HIR* hybrid iterative reconstruction, *SR-DLR* super-resolution deep learning reconstruction, *TP* true positive, *TN* true negative, *FP* false-positive, *FN* false-negative, *PPV* positive predictive value, *NPV* negative predictive value, *AUC* area under the receiver-operator characteristics curve

## Discussion

To the best of our knowledge, this investigation represents the first clinical evaluation of SR-DLR in the ULD setting. The present study demonstrated that ULD SR-DLR achieved 60% dose reduction compared with LD HIR (0.80 ± 0.34 mSv vs. 2.01 ± 0.84 mSv, *p* < 0.001) while offering preserved or even improved image quality. Moreover, ULD SR-DLR performed comparably to LD HIR in coronary plaque and stenosis analysis.

CCTA’s role in the diagnosis, management and risk stratification of CAD is well established [1, 3, 5]. Considering the broadened use of CCTA in clinical practice, the potential radiation risk remains the main concern and stimulates various dose-saving strategies. Meanwhile, advanced reconstruction algorithms have been introduced to compensate for image quality degradation resulting from dose reduction. SR-DLR is a recently commercialized vendor-specific image reconstruction technique for CCTA. To achieve the best possible image quality, the algorithms’ training target was prepared using high-resolution image data acquired on UHRCT scanner (Aquilion Precision) in combination with an advanced deep learning-based noise reduction reconstruction algorithm, while low-quality data with low spatial resolution and high noise content are used as input data. By learning these pairs of target and input data, SR-DLR can yield high-resolution, low-noise images using DCNN.

In this study, between the two ULD datasets, the image noise was significantly lower with SR-DLR than with HIR, and edge sharpness and FWHM of coronary arteries, calcifications and stents were improved by SR-DLR (all *p* < 0.001). These were in accordance with prior studies [18, 22], which found that SR-DLR lowered image noise, improved vessel sharpness, and yielded better delineation of calcification and the stent structures compared with HIR. Given the considerable dose reduction, it was reasonable that the image quality of ULD HIR was inferior to that of LD HIR, with regard to various objective and subjective parameters, whereas the image quality of ULD SR-DLR was non-inferior or even superior to that of LD HIR even at 60% dose reduction. Furthermore, as coronary plaque volume, composition, and stenosis degree are important determinants of patient management and risk stratification [5, 27–29], we tested the feasibility of SR-DLR for radiation dose reduction from these perspectives. On the one hand, coronary plaques were classified into three components (necrotic content, fibrous content or calcified content), and agreements on absolute volumes of all three components were excellent between LD HIR and ULD SR-DLR (ICCs: 0.909, 0.973, 0.980 respectively). Reliability of plaque characterization was also excellent, with ICC of 0.870. This was in accordance with the result of Benz et al [30], which found that a deep learning-based reconstruction method allowed for 43% reduction in radiation dose from CCTA without significant impact on plaque composition and quantitative plaque volume. On the other hand, agreement on stenosis analysis was excellent between LD HIR and ULD SR-DLR, with ICC of 0.973 and using ICA as the reference standard, ULD SR-DLR performed comparably to LD HIR in detecting significant stenosis, with no evidence of a difference in all diagnostic parameters (all *p* > 0.05). Moreover, ICC for per-patient CAD-RADS category was 0.983 between LD HIR and ULD SR-DLR, indicating that dose optimization via SR-DLR has no detrimental effect on patient management. Of note, agreement with stenosis grading from LD HIR was improved with ULD SR-DLR compared with ULD HIR (ICC: 0.973 vs. 0.829) and using ICA as the reference standard, specificity and PPV were also improved (specificity: 83% vs. 52%; PPV: 86% vs. 68%; *p* < 0.05). We believe the reduced image noise and improved edge sharpness by SR-DLR enabled better delineation of vessel border and lesions, thereby leading to more reliable stenosis analysis and confident diagnosis of CAD. This was in line with a prior study [31], which demonstrated that SR-DLR provided superior image quality and diagnostic accuracy for the detection of in-stent stenosis ≥ 50%, in comparison with a model-based IR algorithm. Based on the above results, we can cautiously conclude that SR-DLR achieved 60% dose reduction without compromising image quality, coronary plaque and stenosis severity analysis, which is desirable for CCTA. The superiority of SR-DLR algorithm over HIR well compensated for the detrimental effects that substantial dose reduction could have.

However, several limitations to this study are worth consideration. First, patients underwent two sequential CCTA scans. However, the total ED of LD and ULD scans combined for patients ranged from 0.88 to 5.32 mSv with an average of 2.81 mSv, which is acceptable. Second, the sample size was relatively small, especially the number of patients with ICA as a reference, and data were collected in a single institution. To validate and expand our initial observations, further large-scale multi-center studies are warranted. Third, we did not compare plaque analysis with a reference standard, such as intravascular ultrasound (IVUS), and further research is underway.

## Conclusions

In conclusion, the novel SR-DLR algorithm allows for radiation dose reduction by 60% with preserved or even better image quality. Moreover, SR-DLR ensures excellent performance in the coronary plaque quantification and characterization, and stenosis severity analysis in the ULD scans, which paves the way for its implementation in clinical practice.

## Supplementary information

ELECTRONIC SUPPLEMENTARY MATERIAL

## Abbreviations

CAD: Coronary artery disease
CAD-RADS: Coronary Artery Disease-Reporting and Data System
CCTA: Coronary CT angiography
ED: Effective dose
HIR: Hybrid iterative reconstruction
ICA: Invasive coronary angiography
LD: Low dose
SR-DLR: Super-resolution deep learning reconstruction
ULD: Ultra-low-dose
