# Supplementary material for: Ultra-low-dose coronary CT angiography via super-resolution deep learning reconstruction: impact on image quality, coronary plaque, and stenosis analysis
Source: Eur Radiol. 2025 Feb 1;35(8):4674–84. doi: 10.1007/s00330-025-11399-2 (PMC12226659; doi:10.1007/s00330-025-11399-2)
Supplement: Supplementary file 1 — ELECTRONIC SUPPLEMENTARY MATERIAL [file 330_2025_11399_MOESM1_ESM.pdf]

Ultra-low-dose coronary CT angiography via super-resolution deep learning reconstruction: impact on image quality, coronary plaque, and stenosis analysis

ELECTRONIC SUPPLEMENTARY MATERIAL

Table 1. Subjective image quality assessment

	LD HIR	ULD HIR	ULD SR-DLR	*p* values			
				ALL	LD HIR vs. ULD HIR	LD HIR vs. ULD SR-DLR	ULD HIR vs. ULD SR-DLR
Reader 1	3.30 (0.46)	2.52 (0.58)	4.42 (0.54)	<0.001	<0.001	<0.001	<0.001
Reader 2	3.32 (0.47)	2.48 (0.54)	4.54 (0.50)	<0.001	<0.001	<0.001	<0.001

Data are presented as mean ± standard deviation. LD, low dose; ULD, ultra-low-dose; HIR, hybrid iterative reconstruction; SR-DLR, super-resolution deep learning reconstruction.

Table 2. Agreement on per-patient CAD-RADS category

A. Agreement on per-patient CAD-RADS category between LD HIR and ULD HIR

	LD HIR					
ULD HIR	CAD-RADS 1	CAD-RADS 2	CAD-RADS 3	CAD-RADS 4a	CAD-RADS 4b	CAD-RADS 5
CAD-RADS 1	2	0	0	0	0	0
CAD-RADS 2	2	9	0	0	0	0
CAD-RADS 3	0	7	12	0	0	0
CAD-RADS 4a	0	0	3	11	0	0
CAD-RADS 4b	0	0	0	0	2	0
CAD-RADS 5	0	0	0	0	0	2

B. Agreement on per-patient CAD-RADS category between LD HIR and ULD SR-DLR

	LD HIR					
ULD SR-DLR	CAD-RADS 1	CAD-RADS 2	CAD-RADS 3	CAD-RADS 4a	CAD-RADS 4b	CAD-RADS 5
CAD-RADS 1	4	0	0	0	0	0
CAD-RADS 2	0	15	0	0	0	0
CAD-RADS 3	0	1	14	0	0	0
CAD-RADS 4a	0	0	1	11	0	0
CAD-RADS 4b	0	0	0	0	2	0
CAD-RADS 5	0	0	0	0	0	2

CAD-RADS, Coronary Artery Disease-Reporting and Data System; LD, low dose; ULD, ultra-low-dose; HIR, hybrid iterative reconstruction; SR-DLR, super-resolution deep learning reconstruction.
